# Commentary: Can in vitro valve testing reliably predict clinical outcomes?

**DOI:** 10.1016/j.xjon.2022.02.004

**Published:** 2022-02-11

**Authors:** Hugo M.N. Issa, Fraser Rubens

**Affiliations:** a Division of Cardiac Surgery, Heart Institute, University of Ottawa, Ottawa, Ontario, Canada; b School of Epidemiology and Public Health, University of Ottawa, Ottawa, Ontario, Canada

**Figure:**
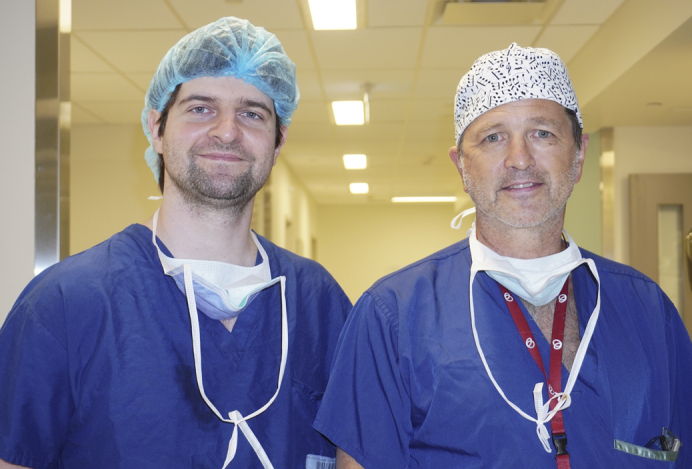
Hugo M. N. Issa, MD, and Fraser Rubens, MD, MSc, FACS, FRCSC

**Central Message**

New generations of aortic valve protheses should always aim at better clinical outcomes, whether physics, hemodynamic, or other parameters also mirror those changes.

See Article page 43.

Hatoum and colleagues^1^ have published an elegant paper showing the hemodynamic parameters and flow characteristics downstream of various transcatheter and surgical aortic valve protheses. They included extensive fluid dynamic considerations by testing the valves in an in vitro environment, using a pulse duplicator. The authors measured the pressure gradient, effective orifice area (EOA), as well as the Reynolds shear stress (RSS) and viscous shear stress (VSS) (those last 2 are used to analyze the degree of turbulence in the downstream flow) among the protheses tested. They demonstrated that the SJM mechanical valve (Abbott Laboratories, Chicago, Ill) had the greatest EOA and that the Hancock II (Medtronic, Minneapolis, Minn) displayed the greatest range of RSS and VSS among the surgical valves. Regarding the transcatheter valves, the Evolut (Medtronic) displayed the greatest range of RSS and VSS compared with the SAPIEN 3 (Edwards Lifesciences, Irvine, Calif). The authors concluded that these features should be considered in the design of future aortic valves prothesis, and that the evolution of the aortic valve protheses have not kept up with improvements in physics.^1^

The obvious question the reader should consider is whether better hemodynamic features translate into better clinical outcomes for patients undergoing aortic valve replacement? Better fluid dynamics do not always predict better clinical outcomes. For example, hemodynamics parameters of the Toronto SPV bioprothesis predicted excellent results; however, the valve was challenged by suboptimum durability, and reoperation was associated with a high complexity.^2^^,^^3^ Similarly, the Hancock II bioprothesis, despite of in vitro predictions of high pressure gradient, RSS, VSS, and low EOA, has demonstrated great durability.^1^^,^^4^^,^^5^ We are not implying that valve hemodynamics in such testing should be ignored; however, findings should be taken in context, and perhaps we should re-examine the implications of laboratory predictions.

We have not yet reached the ideal aortic valve substitute. However, we have been improving clinical outcomes despite a possible lag on the physics features. Today, we can pick an aortic valve prothesis according to the preferences and characteristics of the patient, making each choice unique and individual. Patients at any age are able to choose the valve that best fits them, whether with a surgical or transcatheter approach, whether with a bioprothesis or a mechanical prothesis or a Ross procedure. And, for the future, the new generation of aortic valve protheses should always aim at better clinical outcomes, whether physics, hemodynamic, or other parameters mirror those changes.
